# Radiotherapy in Leptomeningeal Disease: A Systematic Review of Randomized and Non-randomized Trials

**DOI:** 10.3389/fonc.2019.01224

**Published:** 2019-11-15

**Authors:** Samantha M. Buszek, Caroline Chung

**Affiliations:** Department of Radiation Oncology, University of Texas MD Anderson Cancer Center, Houston, TX, United States

**Keywords:** leptomeningeal disease, leptomeningeal carcinomatosis, radiation, neoplastic meningitis, carcinomatous meningitis, systematic (literature) review

## Abstract

**Background:** Leptomeningeal disease (LMD), also known as neoplastic meningitis, leptomeningeal carcinomatosis, or carcinomatous meningitis, is a rare cancer complication occurring in ~5% of cases and ultimately leads to significant morbidity and mortality. In the modern era, incidence of this condition continues to rise with longer survival of patients with advanced and even metastatic disease due to continued improvements in systemic therapies that are providing prolonged control of distant disease, but with limited effect in the central nervous system (CNS). Typical treatment strategies include optimal systemic therapy for the primary disease, as well as neuroaxis directed therapies, which may include intrathecal chemotherapy (ITC) or radiotherapy (RT).

**Methods:** A systematic review of radiotherapy for LMD was performed. Medline, EMBASE, and Cochrane databases were searched from 1946 to 2018 for clinical trials, retrospective/prospective reviews, and case series with ≥2 human subjects that used radiation therapy techniques in the treatment of LMD. The outcome measures of interest included: characteristics of trial participants, inclusion/exclusion criteria, study type, number of participants, primary cancer histology, type of intervention for LMD, survival results if reported, length of follow up, and study conclusion.

**Results:** Of 547 unique citations, 62 studies met the pre-specified eligibility criteria. These studies included 36 retrospective cohorts, 11 prospective series, 12 case series, and a single citation of guidelines, NCDB analysis, and a randomized control trial. Owing to study heterogeneity, meta-analyses of the endpoint data could not be performed.

**Conclusions:** LMD is a devastating complication of cancer with reported survivals ranging from 2 to 4 months. Based on this systematic review, the recommendation for the treatment of LMD is for multimodality discussion of cases and treatment, including the use of radiotherapy, for LMD. However, with continued advances in systemic therapy as well as imaging advances, the landscape of LMD is evolving rapidly and the role of RT will likely also continue to evolve and advance. There is limited high-quality evidence to guide the optimal use of RT for the treatment of LMD, and there is a great need for prospective, histology specific investigation of the role of radiotherapy for LMD in the era of modern systemic therapies.

## Introduction

### Rationale

Leptomeningeal disease (LMD), also known as neoplastic meningitis, leptomeningeal carcinomatosis, or carcinomatous meningitis, is a rare cancer complication in which malignant cells infiltrate the layers of the central nervous system (CNS), known as meninges, and lead to significant morbidity and mortality. This disorder was first diagnosed in 1870, by Eberth et al. and was noted to be a rare complication of malignancy as it was uncommonly diagnosed before death (1). Today, it is known that this condition occurs in ~5% of all cancer patients, presenting most commonly in primary diagnoses of breast cancer (41%), lung cancer (24%), gastrointestinal tract malignancies (13%), and melanoma (12%) (2–4). However, incidence continues to rise with continuous improvements in systemic therapies that allow patients diagnosed with cancer to live longer, as well as improvements in both technology and availability of imaging of the neuroaxis leading to continued increases in the detection and diagnosis of LMD (3).

The anatomy of the neuroaxis consists of the brain and spinal cord, covered by the meninges, which are comprised of dura mater, arachnoid membrane, and pia mater. The leptomeninges refers to the two most inner layers, arachnoid membrane and pia matter, including the subarachnoid space, which separates these two sheets, and is the location of the cerebrospinal fluid (CSF). The CSF is the location of circulating tumor cells in patients with LMD. The pathogenesis of LMD is multifaceted, and can include direct extension from pre-existing CNS tumors or systemic tumors that follow peripheral nerves into the subarachnoid space, as well as infiltration through hematogenous dissemination, or even seeding of the subarachnoid space during surgical procedures (3, 5–8). Once malignant cells gain access to the CSF, they can spread along the meningeal surface by direct extension and be carried by the CSF flow and form new metastatic deposits in other locations.

The clinical presentation of LMD can be widely variable with signs and symptoms of increased intracranial pressure, cranial nerve palsies, radiculopathies, or other focal neurologic deficits, seizures, changes in brain metabolism, or encephalopathy (9–12). A subset of patients present asymptomatic with an incidental imaging finding. Commonly, patients may report a headache or worsening back or radicular pain, as well as signs and symptoms that suggest involvement of multiple anatomic neurologic sites, including: incontinence, lower motor neuron weakness, and sensory abnormalities. In addition to clinical suspicion and signs of LMD on imaging, the most informative diagnostic study is evaluation of the CSF from a lumbar puncture. The opening pressure, cytology, cell count, and measurements of protein and glucose should be performed (13). However, normal lumbar puncture (LP) studies have been reported in 5% of confirmed LMD cases, and, therefore, the requirement of a positive LP is not necessary for diagnosis (14, 15).

Standard treatments for LMD include neuroaxis directed therapies in addition to optimal systemic therapy for the primary and extra-CNS disease. These treatments may include intrathecal chemotherapy (ITC) to target microscopic tumor on the surfaces of the leptomeninges and cells floating in the CSF to prevent further seeding. Radiotherapy (RT) is also commonly used either focally to treat symptomatic sites and areas of bulky disease that will be unlikely to be adequately treated with chemotherapy or in some settings to treat the entire neuroaxis.

### Objectives

Regardless of systemic and local control of the primary disease, prognosis in the setting of LMD is very poor, with reported average survivals of ~2–4 months, even with treatment (16–19). There are currently no standardized guidelines for the treatment of LMD, and many clinicians are hesitant to treat patients with clinically advanced neoplastic meningitis given the high risk of toxicity and unknown value of treatment. Furthermore, in the modern era of immunotherapy, it is unclear if the incidence or prevalence of LMD is increasing.

### Research Question

Therefore, the objective of this systematic review was to examine the outcomes of the use of RT to treat LMD from any primary cancer histology. We reviewed clinical trials, retrospective reviews, and case series with ≥2 subjects that utilized radiation therapy techniques in the treatment of LMD, and report our findings below.

## Methods

### Study Design

The present systematic review was designed to evaluate the published evidence underlying the use of RT treatment for LMD.

### Participants, Interventions, Comparators

The Population Intervention Comparator Outcome Study (PICOS) design framework was used to structure the research question for the review and the corresponding published data search. The population of interest included patients of any age with a diagnosis of LMD who had RT as a part of their treatment regimen once LMD was diagnosed. No restrictions in terms of age, comorbidities, previous treatment received, or method of diagnosis were used. Patients must have received RT as a part of their LMD treatment, but were allowed to receive any other treatment in addition to RT. All doses, preparations, and frequencies of administration were considered. The primary outcome of interest was overall survival (OS). All study types were permitted, including randomized and non-randomized interventional studies, observational studies, and case series with ≥2 human subjects. Only studies with full articles published in English, or with an English translation were considered.

### Systematic Review Protocol

The report documenting our systemic review as prepared in consultation with the Preferred Reporting Items for Systemic Review and Meta-Analysis (PRISMA) statement for systematic review and meta-analyses (20).

### Search Strategy

An information specialist (G.P.) designed and executed an electronic data search to seek relevant citations for the present systematic review from Medline (1946-present), EMBASE (1947-present), and Cochrane (all years) databases. The last search was completed on 7/30/2018. The following search terms to search all trials registers and databases were used: randomized controlled trial (RCT), controlled clinical trial, leptomeningeal carcinomatosis, leptomeningeal metastasis, neoplastic meningitis, meningeal metastasis, meningeal carcinomatosis, radiation therapy, radiotherapy, radiation. A full summary of the search strategies used is provided in Supplementary Appendices 1–3.

### Data Sources, Studies Sections, and Data Extraction

Stage 1 screening consisted of an independent review of the titles and abstracts by two reviewers (S.B., and C.C.). Discrepancies between studies identified as potentially eligible were reconciled between the two reviewers by consensus. Stage 2 screening consisted of an independent, full-text review of all potentially relevant articles by two reviewers. Data collection of the final articles was performed by reviewer S.B. using a standardized data extraction template. The following information was collected: characteristics of trial participants that had LMD, the trials inclusion/exclusion criteria, study type (retrospective, prospective, randomized controlled trial), number of participants treated with RT for LMD, primary cancer histology, type of intervention, survival results if reported, length of follow up, and study conclusion.

### Data Analysis

Due to limitations in the available data, the number of prospective series and randomized controlled trials, and variation in treatments and outcome reporting, it was not possible to perform a detailed meta-analysis. A descriptive approach to summarizing the data was therefore used.

## Results

### Available Evidence

**Figure 1:**
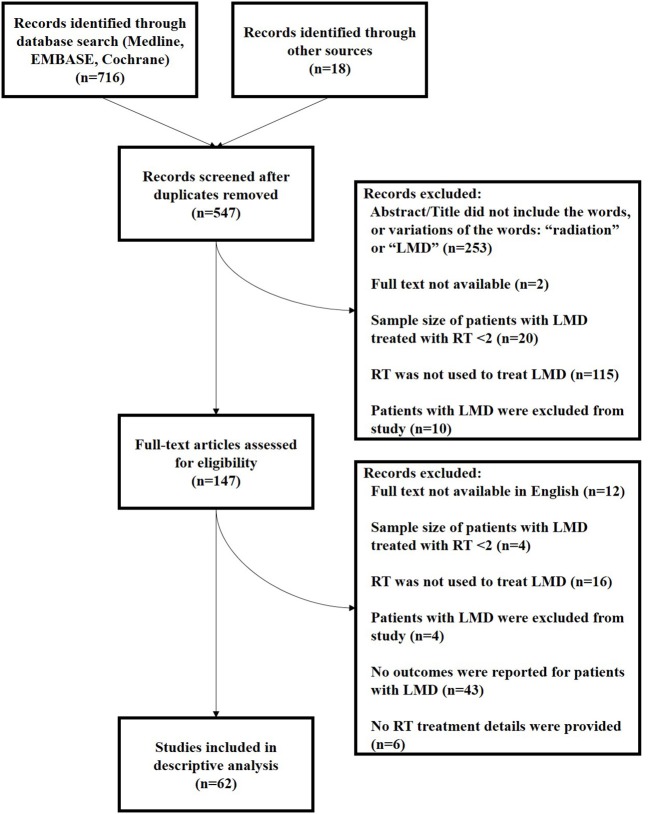
Flow diagram of study selection. N, number of studies; LMD, leptomeningeal disease or other variations of the term; RT, radiation therapy.

A consort diagram of the study selection process is shown in Figure 1. The search of Medline, EMBASE, and Cochrane provided a total of 716 citations; 18 additional citations were identified through other sources including Google and Pubmed. After adjusting for duplicates, 547 citations were reviewed, of which 400 studies were discarded for the following reasons: 253 studies did not include the words or variations of the words: “radiation” or “leptomeningeal disease” in the title or abstract, and thus they were deemed unlikely to be the focus of the publications, 2 citations had no traceable abstract or full text available, and 20 manuscripts reported on only 1 patient with LMD treated with RT. Of the remaining citations, 115 citations did not evaluate the use of RT treatment for LMD and 10 citations excluded patients with LMD and therefore, these 125 citations were excluded. An additional 147 full text articles were subsequently reviewed for inclusion. Of these, 85 were excluded for the following reasons: 12 citations did not have a full text available in English or an English translation; four full texts were found to have a single patient treated with RT for their LMD, thus not meeting inclusion criteria; 16 citations did not evaluate the use of RT to treat LMD; four citations excluded patients with LMD from their study; 43 manuscripts did not report the outcomes of patients with LMD treated with RT; and finally, six studies did not include any details of the RT treatment, and were excluded. Therefore, a total of 62 studies met inclusion criteria and were included in the systematic review.

### Study Selection and Characteristics

The included studies were first categorized according to the primary histology included, and then by the type of study (randomized, observational [retrospective review, prospective review, and case series], guidelines, NCDB analyses, and practice patterns). The results of the following histologies are detailed, below: mixed, breast cancer, non-small cell lung cancer, gastrointestinal (GI) cancer, adult central nervous system (CNS) gliomas, melanoma, leukemia/lymphoma, gynecologic cancer, esthesioneuroblastoma, pediatric CNS disease, and pediatric rhabdomyosarcoma.

### Synthesized Findings by Histology

#### Mixed Histologies

A total of 19 studies reported on patients with LMD from a mix of histologies (Table 1) (15, 17, 21–36, 38). Eighteen of these studies were observational in nature and included patients with breast (*n* = 375), melanoma (*n* = 68), lung (*n* = 469), bladder (*n* = 2), prostate (*n* = 1), ovarian (n = 3), solid tumors not otherwise specified (NOS; *n* = 59), gastrointestinal NOS (*n* = 12), colon (*n* = 2), gastric (*n* = 12), kidney (*n* = 2), adult brain tumors (*n* = 51), unknown primary (*n* = 69), leukemia/lymphoma (*n* = 111), and pediatric brain tumors (*n* = 6). The median age of these patients ranged from 13 to 65 years. Patients were treated with any combination of whole brain radiotherapy (WBRT), craniospinal irradiation (CSI), focal brain RT (FBRT; fractionated or stereotactic radiosurgery [SRS]), focal spine radiotherapy (FSRT), intrathecal chemotherapy (ITC), intraventricular radioisotope, systemic chemotherapy (C), and best supportive care (BSC). Reported OS for the cohorts ranged from 1.4 to 10 months. Additionally, Coakham et al. reported their use of I-131 radiolabeled monoclonal antibodies to treat neoplastic meningitis in pediatric and adult patients between 1984 and 1993 and reported a mean survival of 39 months in therapy responders and 4 months in non-responders (35). These results are sufficiently encouraging to stimulate further attempts at cerebrospinal fluid therapy with I-131 monoclonal antibodies; however, since this time, there is very little reported use of this technique in the treatment of LMD in the literature. Overall, the included studies involving mixed histologies leading to LMD reported a variety of conclusions, with an overall consensus that any treatment had a lower risk of death than supportive care alone. Patients who were able to complete a course of WBRT lived longer, and those who received combined modality treatment had the longest survival.

**Table 1:** Mixed histologies: Included studies evaluating the use of RT for LMD.

**References**	**Study type**	**Study Pop with LMD (*n*)**	**Treatment era (years)**	**Median age, years**	**Treatment**	**Median OS (months)**	**Conclusions**
Passarin et al. (21)	Obs	50	2005–2010	50	48% BSC; 22% C; 6% ITC; 20% RT; 4% combined	1.4	Male gender and any tx favored survival
Gani et al. (22)	Obs	27	2004–2010	57	100% WBRT	2	Presence of CN deficit favored worse OS WBRT alone is effective and feasible if low PS or unfit for other tx
Brower et al. (23)	Obs	124	1999–2014	52	47.2% WBRT; 31.4% C; 7.4% ITC	2.3	C+WBRT favored survival
Clarke et al. (24)	Obs	187	2002–2004	56.4	55% RT; 29% ITC; 18% C; 21% BSC	2.4	PS and tumor type predicted OS Hematopoietic tumors fare better than solid tumors
Oechsle et al. (25)	Obs	135	1989–2005	54	28% C+IT C; 22% ITC; 12% RT + ITC; 7% other; 13% BSC	2.5	Age <50, low PS, <12 months between dx and LMD, lung/melanoma, lack of CSF response have worse OS C leads to longer survival
Kwon et al. (26)	Obs	80	2004–2011	54	90% ITC; 70.9% WBRT; 17.5% FSRT	2.7	Combined modality treatment should be considered
Hermann et al. (17)	Obs	16	1995–2000	46	100% CSI	3	68% regression of symptoms after RT No late toxicity CSI is feasible and effective
Sause et al. (27)	Obs	26	1981–1985	54	IT C + WBRT	3.1	Overall prognosis is poor Aggressive tx may be indicated in some
Chamberlain et al. (28)	Obs	40	1986–1995	56.5	55% WBRT; 17.5% FSRT	4	Bulky metastatic CNS disease predicts survival
Herrlinger et al. (29)	Obs	155	1980–2002	53	10% RT; 32% C; 31% RT+C; 17% BSC	4.8	UVA: >60 years, elevated CSF albumin or lactate predicted poor survival C alone or in combo may improve outcomes
Wasserstrom et al. (15)	Obs	90	1975–1980	NR	73% RT; 100% ITC	5.8	Radiosensitive tumors: CSI is very effective
Chamberlain et al. (30)	Obs	15	1987–1994	13	100% C and ITC; 60% RT	6	Children with hematologic malignancies had superior outcomes to solid tumors RT of bulky dz is indicated
Sakaguchi et al. (31)	Obs	206	2000–2015	65	100% WBRT; 8% surgery	6	MVA: high PS, asymptomatic, favorable primary lesion, surgery+WBRT favored survival
Pan et al. (32)	Obs	59	2010–2014	55	50% ITC+WBRT; 86% WBRT; 34% FSRT; 25% WBRT+FSRT; 71% BSC	6.5	MVA: extensive disease, lung favored poor prognosis Focal RT+ITC improves quality of life and symptoms
Bokstein et al. (33)	Obs	104	1998	52	40%WBRT; 12% FSRT; 14% WBRT+FSRT; 6% CSI;4% C	7	ITC had higher complication rates
Wolf et al. (34)	Obs	32	2013–2015	62	100% SRS	10	Focal LMD may be treated with SRS, delaying WBRT
Coakham et al. (35)	Obs	7	1984–1993	42	100% I-131 MAb	Mean survival responders: 39; non-responders :4	Results are promising, should consider further studies with MAb
Du et al. (36)	Obs	46	2008–2011	53	26% BSC; 57% C; 47% RT; 11% ITC	4.4	Prolonged OS in NSCLC pts receiving TKIs High PS, WBRT or focal RT + C improve OS
Hyun et al. (37)	NCDB	519	2005–2014	56	28% BSC; 45% C; 10% RT; 17% C+RT	5	Most pts have poor outcomes UVA: young, Female, low CSF protein, high PS, active tx improved survival

*RT, radiotherapy; LMD, leptomeningeal disease; Pop, population; Obs, observational study; NR, not reported; BSC, best supportive care; C, chemotherapy alone; ITC, intrathecal chemotherapy; WBRT, whole brain radiotherapy; FSRT, spine RT; CSI, craniospinal RT; MAb, monoclonal antibody; Tx, treatment; PS, performance status; dz, disease; SRS, stereotactic radiosurgery; TKI, tyrosine kinase inhibitor*.

A National Cancer Database (NCDB) analysis reported the results of patients treated for LMD from any histology from 2005 to 2014. Patients were diagnosed with lung cancer (*n* = 334), breast cancer (*n* = 96), GI cancer NOS (*n* = 39), leukemia/lymphoma (*n* = 19), and unknown primary (*n* = 3). Of the cohort, 52 patients were treated with RT alone, 88 patients were treated with RT+C, 232 patients were treated with C alone, and 147 patients were treated with BSC. Of the patient's that received RT, 85% were treated with WBRT, 14% with focal brain RT (FBRT), and 1% with WBRT and focal spine RT (FSRT). Median OS for the entire cohort was 3 months (2.7–3.3 months). Treated patients exhibited a higher median OS compared to BSC; and patients treated with C+RT revealed the highest median OS of 5 months (3.5–6.5 months) followed by patients treated with RT alone who had a median OS of 3 months (1.7–4.3 months). The authors concluded that patients with LMD have poor outcomes overall, but those who had a good performance status and normal CSF levels had a more favorable prognosis upon active treatment (37).

#### Breast Cancer

Nine studies meeting inclusion criteria included patients with a primary histology of breast cancer (Table 2) (39–48). Of these, 7 studies reported outcomes of observational studies, and a single study reported the results of guideline recommendations, and a randomized controlled trial. The observational studies included a total of 764 patients treated with RT for LMD in sample sizes ranging from 8 to 187 per study. These patients were treated from 1986 to 2015 and ranged in age from 23 to 80 years. Patients were treated with WBRT (*n* = 243), FSRT (*n* = 47), C (*n* = 220), ITC (*n* = 434), or BSC (*n* = 20), with multiple patients receiving combinations of these treatments; however, exact numbers were not reported. The OS for patients treated on these studies ranged from 3.8 to 7.5 months. Longer survival was associated with hormone receptor positive tumors, limited prior therapy for systemic disease, and LMD as the site of first metastasis, with mixed conclusions on the impact of improvement when RT is added to C/ITC. A positive correlation was noted between improved quality of life and treatment with RT in the single study that evaluated this endpoint (44). Any treatment was associated with longer survival when compared to BSC alone. Interestingly, a high burden of disease at the time of LMD diagnosis was not found to be associated with worse survival (45). Overall, studies supported inclusion of RT as a part of the multimodality treatment approach in LMD of breast cancer.

**Table 2:** Breast cancer: Included studies evaluating the use of RT for LMD.

**References**	**Study type**	**Study Pop with LMD (*n*)**	**Treatment era (years)**	**Median age, years**	**Treatment**	**Median OS (months)**	**Conclusions**
Boogerd et al. (39)	RCT	35	1991–1998	35	49% ITC; 46% C; 37% HT; 43% RT	4.3 for ITC; 7 for no-ITC	Addition of ITC does not lead to OS benefit or improved neurologic response ITC is associated with increased risk of toxicity
Feyer et al. (40)	G	NA	1995–2008	NA	NA	NA	RT is an effective Tx for LMD CSI generally not recommended
Le Rhun et al. (41, 42)	Obs	103	2007–2011	39	100% ITC; 58% C; 17% RT	3.8	Long survivors: young, ER/PR+, limited prior C, LMD is first site of mets, good PS, no hydrocephalus, bulky disease on imaging, long duration of LMD tx Better OS: multimodality tx for LMD, 2nd line ITC, ER/PR+
Niwinska et al. (43)	Obs	187	1999–2015	49	68% ITC; 63% WBRT; 14% FSRT	4	Better OS: old age, high PS, luminal subtype, C, RT, ITC
Niwinska et al. (44)	Obs	149	1999–2015	49	52% C; 62% RT; 65% ITC	4.2	C+RT is 2x stronger factor associated with improved OS than ITC Improved OS: old age, high PS, luminal subtype, C, RT, ITC
Yust-Katz et al. (45)	Obs	103	1995–2011	49.2	19% BSC; 53% WBRT; 19% FSRT; 36% C; 56% ITC	4.3	Any tx leads to improved OS Multimodal tx leads to improved OS Load of systemic dz not associated with worse OS
Kingston et al. (46)	Obs	182	2004–2014	52.5	34% WBRT; 25% C; 7.7% ITC	5.4	C has the longest OS MVA: triple neg, brain mets, LMD of brain and spinal cord has worse OS
Yu et al. (47)	Obs	8	1990–1999	51.5	100% WBRT; 12.5% FSRT; 50% ITC	5.4	Need high suspicious for LMD WBRT + ITC is promising
Chamberlain et al. (48)	Obs	32	1986–1995	49	66% RT; 100% ITC	7.5	Comprehensive CNS evaluation of LMD and aggressive combined modality tx has modest improvement in survival

*RT, radiotherapy; LMD, leptomeningeal disease; Pop, population; Obs, observational study; RCT, randomized controlled trial; G, guidelines; NR, not reported; BSC, best supportive care; C, chemotherapy alone; ITC, intrathecal chemotherapy; WBRT, whole brain radiotherapy; FSRT, spine RT; CSI, craniospinal RT; HT, hormonal therapy*.

Feyer et al. published practical guidelines for palliative RT of breast cancer with brain metastases and LMD (40). The guidelines come from the German Society of Radiation Oncology (DEGRO) after performing a comprehensive survey of the literature. For patients with LMD, the group indicates that treatment is mostly indicated for patients with positive cytology in the CSF or with signs or symptoms of neurologic deficits. Treatment may include RT (WBRT or focal RT), C or ITC, or both—with prolongation of survival from several weeks to 4–6 months.

Boogerd et al. published a randomized controlled trial to determine the relevance of ITC for LMD in breast cancer (39). Patients were randomized to ITC (*n* = 17) or non-ITC treatment (*n* = 18). Appropriate systemic therapy and RT were given in each group (35% of patients in the ITC arm received focal RT and 2 patients received WBRT; 50% of patients in the non-ITC arm received focal RT, and 4 received WBRT). The authors found that standard systemic chemotherapy with focal RT for LMD is feasible; however, the addition of ITC did not lead to a survival benefit or improved neurological response, and was associated with an increased risk of neurotoxicity.

#### Non-small Cell Lung Cancer

A total of eight observational studies of 893 patients treated from 1986 to 2014 for LMD from non-small cell lung cancer were included (Table 3) (52–59). Patients were treated with WBRT (*n* = 389), FSRT (*n* = 10), C (*n* = 39), targeted therapy (TT; *n* = 278), ITC (*n* = 7), or BSC (*n* = 31). Overall survival ranged from 3 to 8.7 months for all patients. There were conflicting findings on whether WBRT or tyrosine-kinase inhibitors (TKIs) improved survival for patients; survival was improved for selected patients who received ITC. Similar to other histologies, patients with good performance status at the time of LMD diagnosis had better outcomes.

**Table 3:** Non-small cell lung cancer: Included studies evaluating the use of RT for LMD.

**References**	**Study type**	**Study Pop with LMD (*n*)**	**Treatment era (years)**	**Median age, years**	**Treatment**	**Median OS (months)**	**Conclusions**
Morris et al. (52)	Obs	125	2002–2009	59	45% WBRT; 6% ITC; 16% C; 15% TT; 30% BSC	3	No difference in OS with WBRT ITC had superior OS
Kuiper et al. (53)	Obs	32	2000–2014	54	78% TT; 6% C; 13% C+TT; 34% WBRT; 9% FSRT	3.1	Good KPS improved OS (not TT, RT, LMD as only site of disease) RT did not affect OS
Lee et al. (54)	Obs	149	2001–2009	58	32% WBRT; 24% C; 13.4% BSC	3.5	MVA: low PS, high CSF counts predicted poor OS; ITC, TT, WBRT predicted improved OS
Ozdemir et al. (55)	Obs	51	2007–2014	53	100% WBRT	3.9	Beneficial role for WBRT, especially if good PS, time to LMD >11.3 mo, no brain mets at presentation
Liao et al. (56)	Obs	212	2003–2010	56	58.5% TT; 60.4% WBRT	4.5	MVA: TT, WBRT, C improve survival
Chamberlain et al. (57)	Obs	32	1986–1996	57	28% WBRT; 22% FSRT	5	Comprehensive evaluation of extent of dz and aggressive combined modality tx leads to modest improvement in OS
Xu et al. (58)	Obs	108	2006–2013	61	45% WBRT; 39% TT	5.3	Longest OS in TT+WBRT MVA: good KPS, WBRT, TT improved OS
Li et al. (59)	Obs	184	2011	57	45% TT; 5.5% WBRT; 0.9% C; 38.5% combined tx; 10% BSC	8.7	Longest OS in TT alone (vs WBRT alone or WBRT +TT) MVA: TT improved OS and poor KPS worsened OS

*RT, radiotherapy; LMD, leptomeningeal disease; Pop, population; Obs, observational study; PP, practice patterns; NR, not reported; BSC, best supportive care; C, chemotherapy alone; ITC, intrathecal chemotherapy; WBRT, whole brain radiotherapy; FSRT, spine RT; CSI, craniospinal RT; TT, targeted therapy*.

#### Gastrointestinal Cancer

Seven observational studies incorporated patients with gastrointestinal (GI) malignancies and LMD (Table 4) (60–66). These studies included 127 patients treated from 1944 to 2010 with the diagnoses of gastric cancer (*n* = 99), esophageal cancer (*n* = 7), and GI NOS (*n* = 21). Patients were treated with RT alone, NOS (*n* = 30), WBRT (*n* = 19), FSRT (*n* = 1), ITC (*n* = 15), RT + ITC (*n* = 48), C (*n* = 8), RT+C (*n* = 3), or BSC (*n* = 7). The median OS ranged from 1 to 3 months. From these studies, it was concluded that ITC improved survival on multivariate analysis and cytological negative conversion was predictive of a relatively longer survival; however, there was no clear benefit of the use of RT in patients with LMD from GI malignancies.

**Table 4:** Gastrointestinal cancer: Included studies evaluating the use of RT for LMD.

**References**	**Study type**	**Study Pop with LMD (*n*)**	**Treatment era (years)**	**Median age, years**	**Treatment**	**Median OS (months)**	**Conclusions**
Lee et al. (60)	Obs	19	1992–2002	48	16% WBRT; 5% FSRT; 5% CSI; 53% ITC	1	ITC improved OS
Lukas et al. (61)	Obs	7	NR	60	14% ITC; 57% WBRT	1.2	LMD from esophageal cancer has a poor prognosis
Kim et al. (62)	Obs	5	1985–1992	43	100% WBRT; 40% ITC	1.4	Neither C or WBRT affected clinical course
Oh et al. (63)	Obs	54	1994–2007	48.5	61% ITC, 24% WBRT + ITC; 11% WBRT; 19% C; 13% C+ITC	1.6	MVA: cytological negative conversion predicts longer survival
Giglio et al. (64)	Obs	21	1944–2002	NR	43% RT; 19% ITC; 5% RT+ITC; 5% RT+C; 19% BSC	1.75	Poor outcomes with GI malignancy BSC may be reasonable alternative
Tomita et al. (65)	Obs	12	2002–2009	63	83% ITC; 58% WBRT	2	Multidisciplinary treatment may benefit select pts
Kim et al. (66)	Obs	9	1995–2010	53	33% BSC; 33% RT; 22% C+RT; 11% ITC	3	LMD is extremely fatal in GI cancer High index of suspicion is needed

*RT, radiotherapy; LMD, leptomeningeal disease; Pop, population; Obs, observational study; PP, practice patterns; NR, not reported; BSC, best supportive care; C, chemotherapy alone; ITC, intrathecal chemotherapy; WBRT, whole brain radiotherapy; FSRT, spine RT; CSI, craniospinal RT*.

#### Adult CNS Gliomas

Seven observational studies incorporated patients with LMD from adult gliomas (Table 5) (67–73). These studies incorporated 101 patients treated between 1978 and 2015, with diagnoses of glioblastoma (GBM, *n* = 77), World Health Organization (WHO) grade II glioma (*n* = 1), WHO grade III astrocytoma (*n* = 14), and oligodendrogliomas (*n* = 7). Patients were treated with FBRT (*n* = 5), BSC (*n* = 5), C (*n* = 16), C+CSI (*n* = 4), C+FBRT+FSRT (*n* = 3), C+FBRT (*n* = 2), C+ whole spine RT (*n* = 1), and surgical resection (*n* = 2). The median OS from the diagnosis of LMD ranged from 2.8 to 10.2 months, with 32 months for oligodendrogliomas NOS. The series that included non-GBM's reported the longer survival outcomes. These studies indicated that patients treated with a combination of C+RT had significantly prolonged survival compared to either therapy alone or BSC. Patients with no symptoms from LMD and better performance status at presentation had longer survival with no difference in outcomes between patients with large volume vs. small volume LMD disease.

**Table 5:** Adult CNS gliomas: Included studies evaluating the use of RT for LMD.

**References**	**Study type**	**Study Pop with LMD (*n*)**	**Treatment era (years)**	**Median age, years**	**Treatment**	**Median OS (months)**	**Conclusions**
Vertosick et al. (67)	Obs	11	1978–1990	38.5	82% FSRT; 9% FBRT; 9% CSI	2.8	LMD occurs in younger pts and pts with extended survival
Mandel et al. (68)	Obs	36	2006–2012	44	14.7% BSC; 6% RT; 47% C; 29% C+RT; 3% surgery	3.5	UVA: combo tx leads to prolonged OS
Cohen et al. (69)	Obs	3	2002	37	100% RT	4	Focused RT should be considered because of its significant therapeutic effect
Burger et al. (70)	Obs	9	2008–2015	40.8	78% C; 11% WBRT; 44% FSRT; 33% FBRT	4.3	Addition of bevacizumab is a novel treatment option with good therapeutic effects in brain
Endo et al. (71)	Obs	5	1997–2001	38	80% surgery+RT+C; 20% SRS	7.6	Tx with C+RT may be required
Dardis et al. (72)	Obs	34	2003–2013	49	26% ITC; 44% RT; 62% C; 9% surgery	10.2	Benefit of RT in LMD Consider pts KPS
Roldan et al. (73)	Obs	8	1991–2009	41	62.5% BSC; 12.5% RT; 12.5% C; 12.5% C+RT	32	LMD in oligodendrogliomas is indolent

*RT, radiotherapy; LMD, leptomeningeal disease; Pop, population; Obs, observational study; PP, practice patterns; NR, not reported; BSC, best supportive care; C, chemotherapy alone; ITC, intrathecal chemotherapy; WBRT, whole brain radiotherapy; FSRT, spine RT; CSI, craniospinal RT*.

#### Melanoma

Three observational studies reviewed patients with a diagnosis of LMD from melanoma (Table 6) (49–51). These authors reviewed 140 patients treated from 1944 to 2015 treated with FSRT (*n* = 26), FBRT (*n* = 2), WBRT (*n* = 42), FSRT+FBRT (*n* = 9), C (*n* = 54), targeted therapy (TT; *n* = 9), ITC (*n* = 53), and surgical resection (*n* = 3); with 9 patients receiving TT+RT treatments. The median OS ranged from 2.3 to 5.2 months with the longest survival seen in the most modern series that included immunotherapy (50). The median OS for the 9 patients treated with TT+RT was 7.2 months. These studies found that survival is improved with the introduction of anti-PD-1 antibodies and BRAF inhibitors, and that multimodality therapy combined with these new techniques may be required to obtain the best control of neurologic symptoms and improve survival. Unfortunately, given the timeline of treatment of patients on these studies, only was study was performed in the modern era of immunotherapy and targeted therapy.

**Table 6:** Melanoma: Included studies evaluating the use of RT for LMD.

**References**	**Study type**	**Study Pop With LMD (*n*)**	**Treatment Era (years)**	**Median Age, years**	**Treatment**	**Median OS (months)**	**Conclusions**
Chamberlain et al. (49)	Obs	16	1986–1995	47	44% FSRT; 63% WBRT; 75% C	4	Limited survival despite aggressive CNS directed therapies
Arasaratnam et al. (50)	Obs	14	2012–2015	49.8	36% FSRT; 14% SRS; 50% WBRT; 57% TT; 14% surgery; 61% TT+RT	5.2	Modern melanoma therapies can result in symptom improvement with occasional longer survivals; although prognosis is generally still poor Multimodality tx with surgery+RT+TT may be required to prolong survival
Harstad et al. (51)	Obs	110	1944–2002	NR	56% WBRT; 33% FSRT; 27% WBRT+FSRT; 38% C; 48% ITC	2.3	MVA: primary melanoma on trunk has shorter OS; ITC has longer survival

*RT, radiotherapy; LMD, leptomeningeal disease; Pop, population; Obs, observational study; PP, practice patterns; NR, not reported; BSC, best supportive care; C, chemotherapy alone; ITC, intrathecal chemotherapy; WBRT, whole brain radiotherapy; FSRT, spine RT; CSI, craniospinal RT*.

#### Leukemia/Lymphoma

Two observational series met inclusion criteria and were included in the study (Table 7) (74, 75). A total of 47 patients treated between 1988 and 2016 included diagnoses of chronic lymphocytic leukemia (CLL; *n* = 3) and diffuse large B-cell lymphoma (DLBCL; *n* = 44). Patients were treated with FBRT (*n* = 4), WBRT (*n* = 42), and CSI (*n* = 1). The three patients with CLL had an OS of 8 months, 3 months, and still alive at censorship at 6 months. The median OS of the patients with DLBCL was 7 months. These studies indicated that patients with CLL and LMD involvement of their optic nerve had improved survival with focal RT and that RT provides high response rates in DLBCL and can contribute to long-term disease free survival.

**Table 7:** Leukemia/Lymphoma: included studies evaluating the use of RT for LMD.

**References**	**Study type**	**Study Pop with LMD (*n*)**	**Treatment era (years)**	**Median age, years**	**Treatment**	**Median OS (months)**	**Conclusions**
Currie et al. (74)	Obs	3	1988	63	100% FBRT	NR	RT leads to considerable clinical improvement
Milgrom et al. (75)	Obs	44	2006–2016	NR	95% WBRT; 2% CSI; 70% C	7	RT has high response rates

*RT, radiotherapy; LMD, leptomeningeal disease; Pop, population; Obs, observational study; PP, practice patterns; NR, not reported; BSC, best supportive care; C, chemotherapy alone; ITC, intrathecal chemotherapy; WBRT, whole brain radiotherapy; FSRT, spine RT; CSI, craniospinal RT*.

#### Gynecologic Cancer

Two observational studies included patients with gynecologic cancer leading to a diagnosis of LMD (Table 8) (76, 77). These studies included 15 patients treated between 1996 and 2010 with diagnoses of ovarian cancer (*n* = 12) and cervical/endometrial cancer (*n* = 3). Patients were treated with WBRT (*n* = 8), FBRT (*n* = 1), FSRT (*n* = 5), C (*n* = 1), and BSC (*n* = 1). The median overall survival was 3.6 months for patients with ovarian cancer, and 2, 4, and 7 months for the three patients with cervical/endometrial cancer. It was concluded that RT can provide a partial or complete response of LMD, although with recurrence likely.

**Table 8:** Gynecologic cancer: Included studies evaluating the use of RT for LMD.

**References**	**Study type**	**Study Pop with LMD (*n*)**	**Treatment era (years)**	**Median age, years**	**Treatment**	**Median OS (months)**	**Conclusions**
Teckie et al. (76)	Obs	12	1996–2010	56.1	58% WBRT; 8% partial brain RT;17% FSRT; 8% C; 8% BSC	3.6	RT leads to partial or complete response of LMD, but will likely recur or progress
Asensio et al. (77)	Obs	3	2000	63	33% ITC; 100% FSRT; 33% WBRT; 33% C	NR	CSF evaluation may not be sufficient for diagnosis Improving diagnosis may improve outcomes

*RT, radiotherapy; LMD, leptomeningeal disease; Pop, population; Obs, observational study; PP, practice patterns; NR, not reported; BSC, best supportive care; C, chemotherapy alone; ITC, intrathecal chemotherapy; WBRT, whole brain radiotherapy; FSRT, spine RT; CSI, craniospinal RT*.

#### Esthesioneuroblastoma

One observational study met inclusion criteria and included patients with esthesioneuroblastoma (Table 9) (78). In this study, Chamberlain et al. included 4 patients ranging in age from 47 to 58 years treated in 2002 with FSRT (*n* = 2), WBRT (*n* = 3), and C (*n* = 4). The patients had OS of 4, 11, 12, and 13 months, respectively. The authors concluded that LMD from esthesioneuroblastoma is treatable with acceptable toxicity.

**Table 9:** Esthesioneuroblastoma: included studies evaluating the use of RT for LMD.

**Reference**	**Study type**	**Study Pop with LMD (*n*)**	**Treatment era (years)**	**Median age, years**	**Treatment**	**Median OS (months)**	**Conclusions**
Chamberlain et al. (78)	Obs	4	2002	53	50% FSRT; 50% WBRT + ITC	NR	Treatment has acceptable toxicity and reasonable disease palliation

*RT, radiotherapy; LMD, leptomeningeal disease; Pop, population; Obs, observational study; PP, practice patterns; NR, not reported; BSC, best supportive care; C, chemotherapy alone; ITC, intrathecal chemotherapy; WBRT, whole brain radiotherapy; FSRT, spine RT; CSI, craniospinal RT*.

#### Pediatric CNS Disease

Three observational studies met inclusion criteria that included pediatric patients with central nervous system (CNS) disease (Table 10) (79–81). These series evaluated 34 patients (ages 1–34) treated from 1977 to 2012 with diagnoses of astrocytoma (*n* = 6), medulloblastoma (*n* = 9), ependymoma (*n* = 3), atypical teratoid rhabdoid tumor (ATRT; *n* = 4), primitive neuroectodermal tumor (PNET; *n* = 3), and other (*n* = 9). Patients received treatment for their LMD with FSRT (*n* = 28), CSI (*n* = 6), and FBRT (*n* = 6). The median OS was not reached in two studies. In patients with astrocytoma receiving FSRT, the median OS was 7 months. In patients with either medulloblastoma, ependymoma, PNET, or other treated with FSRT, the 12 month OS was 68%; patients with PNET had a median OS of 9.2 months. For patients with primary CNS disease, NOS, the mean OS was 25.7 months with salvage CSI. Therefore, these studies revealed that RT in the treatment of LMD is beneficial with minimal toxicity.

**Table 10:** Pediatric CNS disease: Included studies evaluating the use of RT for LMD.

**References**	**Study type**	**Study Pop with LMD (*n*)**	**Treatment era (years)**	**Median age, years**	**Treatment**	**Median OS (months)**	**Conclusions**
Kandt et al. (79)	Obs	6	1977–1982	9	67% FSRT; 17% C; 17% BSC	7	RT is beneficial
Ray et al. (80)	Obs	22	2004–2012	5	100% FSRT	NYR	Durable response can be achieved with proton RT
Wei et al. (81)	Obs	6	2007–2012	6.5	100% WBRT; 17% CSI; 17% SRS	NR	Salvage CSI is effective

*RT, radiotherapy; LMD, leptomeningeal disease; Pop, population; Obs, observational study; PP, practice patterns; NR, not reported; BSC, best supportive care; C, chemotherapy alone; ITC, intrathecal chemotherapy; WBRT, whole brain radiotherapy; FSRT, spine RT; CSI, craniospinal RT; SRS, stereotactic radiosurgery; NYR, not yet reached; PFS, progression free survival*.

#### Pediatric Rhabdomyosarcoma

One observational series met inclusion criteria and was included with patients with rhabdomyosarcoma and LMD (Table 11) (82). The authors included 13 pediatric patients with LMD (age from 1 to 34 years) treated from 1999 to 2016 with FBRT (*n* = 1), WBRT (*n* = 5), CSI (*n* = 10), C (*n* = 4), surgery (*n* = 2). The median OS was 5 months and the study concluded that treatment with CNS directed RT should be considered, however, outcomes were poor.

**Table 11:** Pediatric rhabdomyosarcoma: Included studies evaluating the use of RT for LMD.

**Reference**	**Study type**	**Study Pop with LMD (*n*)**	**Treatment era (years)**	**Median age, years**	**Treatment**	**Median OS (months)**	**Conclusions**
De et al. (82)	Obs	21	1999–2016	15	48% CSI; 14% C; 19% WBRT; 5% ITC; 14% surgery; 5% SRS	5	Conformal RT techniques, such as proton CSI, may help limit overlap of RT fields and reduce toxicities

*RT, radiotherapy; LMD, leptomeningeal disease; Pop, population; Obs, observational study; PP, practice patterns; NR, not reported; BSC, best supportive care; C, chemotherapy alone; ITC, intrathecal chemotherapy; WBRT, whole brain radiotherapy; FSRT, spine RT; CSI, craniospinal RT*.

#### Ongoing Trials

We performed a search of the ClinicalTrials.gov database to identify any ongoing therapeutic trials utilizing radiotherapy in the treatment of LMD. As of May 2019, we identified 5 ongoing relevant studies (Table 12). One study is using proton radiotherapy alone to the CSI for patients with LMD (84). Three of the trials are evaluating the use of radiotherapy with another drug intervention (pemetrexed, Avelumab, methotrexate, cytarabine) (83, 85, 86). Finally, one trial is evaluating the use of radiation iodine I-131 monoclonal antibody for the treatment of LMD (87).

**Table 12:** Ongoing trials: Evaluating the use of RT for LMD.

**References**	**Study type**	**Study Pop/Pt treated with RT (*n*)**	**Treatment**	**ClinicalTrials.gov Identifier**
H. Lee Moffitt Cancer Center (83)	Phase I	LMD	Arm 1: Avelumab + WBRT	NCT03719768
Memorial Sloan Kettering (84)	Single arm, prospective	LMD	Proton CSI	NCT03520504
The First Hospital of Jilin University Changchun, China (85)	Phase I/II	LMD from solid tumors	ITC (pemetrexed)+RT	NCT03507244
The First Hospital of Jilin University Changchun, China (86)	Phase II	LMD from solid tumors	ITC (methotrexate and cytarabine)+RT	NCT03082144
Memorial Sloan Kettering (87)	Phase I	LMD	I-131 monoclonal Ab RT	NCT00089245

*RT, radiotherapy; LMD, leptomeningeal disease; Pop, population; Obs, observational study; PP, practice patterns; NR, not reported; BSC, best supportive care; C, chemotherapy alone; ITC, intrathecal chemotherapy; WBRT, whole brain radiotherapy; FSRT, spine RT; CSI, craniospinal RT*.

### Risk of Bias

Due to the small number of randomized trials found, all trials that met eligibility criteria were included.

## Discussion

### Summary of Main Findings

**Figure 2:**
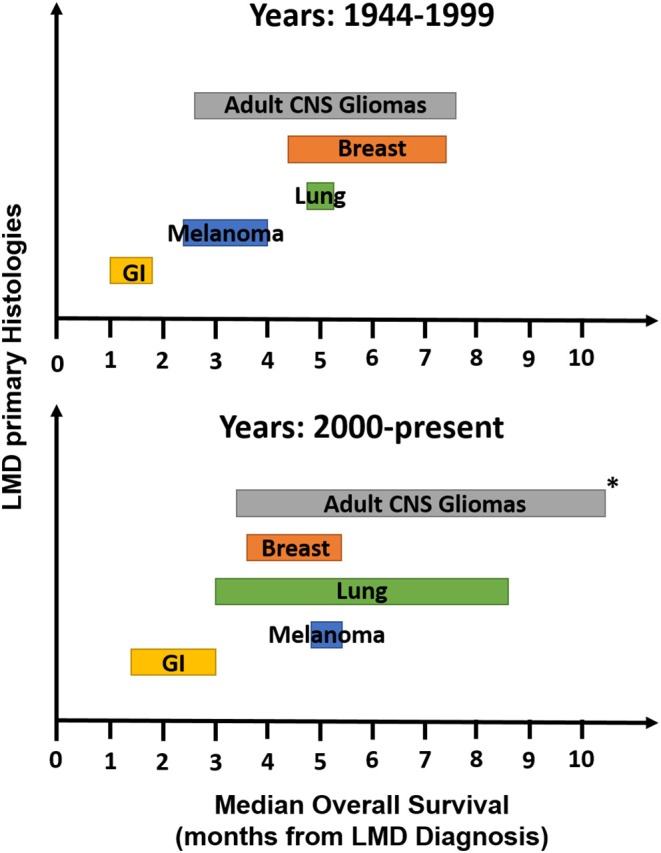
Median OS ranges from observational studies meeting inclusion criteria for the most common histologies of LMD. OS, overall survival; LMD, leptomeningeal disease. *Excluding trial by Roldan et al. (73) which included only patients with oligodendrogliomas and reported an OS of 32 months.

Leptomeningeal disease is an important, but devastating complication of cancer that leads to significant morbidity and mortality. Since its initial discovery in the 1800s, the incidence of this condition has continued to increase as our methods of diagnosis continue to improve and patients with various cancer histologies are experiencing longer survival, even with metastatic disease. Based on the single histology studies included in this systematic review, the incidence of LMD treated with RT from most common to least common was: lung (*n* = 893) > breast (*n* = 799) > melanoma (*n* = 140) > GI (*n* = 127) > adult CNS gliomas (*n* = 106), which is similar to the reported frequencies of the incidence of LMD in all patients, in the literature (2–4). The studies included in this review included patients treated from 1944 to 2015, and all of these studies were included given the small number of studies meeting inclusion criteria in the more modern era. The imaging (or lack thereof) and treatment techniques utilized in the older studies do not reflect how we think about LMD today. Therefore, we have separated these studies into those that included a majority of patients treated with RT prior to the year 2000 and those more modern studies that incorporated a majority of patients treated with RT in the year 2000 and after when evaluating the incidence of LMD treated with RT and the OS seen in these studies, as these outcomes have evolved over time. Prior to the year 2000, the incidence of LMD treated with RT is as follows: melanoma (*n* = 126) > breast (*n* = 75) > GI (*n* = 45) > lung (*n* = 32) > adult CNS gliomas (*n* = 16). After the year 2000, the incidence of LMD treated with RT is as follows: lung (*n* = 861) > breast (*n* = 724) > adult CNS gliomas (*n* = 90) > GI (*n* = 82) > melanoma (*n* = 14). The median overall survival ranges reported in these studies have also evolved over time, and are demonstrated in Figure 2. Prior to the year 2000, OS in patients with LMD treated with RT increased from shortest to longest in primary histologies of GI (1–1.75 months) < melanoma (2.3–4 months) < lung (5 months) < breast (5.4–7.5 months) < adult CNS gliomas (2.8–7.6 months). After the year 2000, OS increased from shortest to longest in GI (1.2–3 months) < breast (3.8–5.4 months) < melanoma (5.2 months) < lung (3–8.7 months) < adult CNS gliomas (3.5–10.2 months).

The optimal management of this condition remains unknown, with limited data to guide standard of care treatment. Current management approaches include a range of treatments including systemic therapy (intravenous or intrathecal), targeted therapy, focal or non-focal radiotherapy, and supportive care. A recent survey study of oncologists across Europe evaluated their diagnosis and treatment patterns for patients with LMD from solid tumors. A total of 115 physicians submitted responses (19% radiation oncologists, 23% medical oncologists, 34% neuro-oncologists, and 10% neurosurgeons) reporting that only 31.5% always administer systemic treatment when feasible, 15.5% felt that WBRT should always be performed, while 73% of medical oncologists, 56% of neuro-oncologists, 50% of radiation oncologists, and 39% of neurosurgeons felt that WBRT should be offered in the setting multifocal nodular disease only. A total of 73% of respondents declared that focal RT should only be performed in cases of neurologic symptoms only when they could be linked to an MRI abnormality and not for neurological signs or symptoms alone (88). This wide range in practice patterns highlights the need for more formal guidelines and recommendations for the treatment of LMD.

Recent developments, to assist in the standardization of diagnosis of LMD, were performed by an international panel of experts from the United States and Europe, termed the RANO group. This group recently published their results of using an MRI scorecard to assess response to treatment in patients with leptomeningeal disease. Unfortunately, after MRIs of 22 patients with LMD were scored by neuro-oncologists and neuroradiologists, many raters experienced difficulty with the instructions of the scorecard and no acceptable alpha concordance coefficient was obtained. The authors are currently working on a more simplified scorecard that will require validation, but will be a great step forward in the standardization of this disease process, hopefully leading to more comparable trials in the future (89).

The National Comprehensive Cancer Network (NCCN) has recently incorporated risk stratification and guidelines for the treatment of LMD (90). Patients with LMD can be defined as Good Risk if they have all of the following characteristics: Karnofsky Performance Status (KPS) ≥60, no major neurologic deficits, minimal systemic disease, and if they have a reasonable systemic treatment options should they need them. Patients in this category are typically diagnosed with LMD based on routine imaging and are being evaluated on an outpatient basis. Ultimately, the level of aggressiveness of the treatment provided should include a discussion with the patient and their wishes. RT is typically included in the multimodal treatment of patients with Good Risk LMD. In contrast to Good Risk LMD, patients with LMD can be defined as Poor Risk if they have any of the following characteristics: KPS <60, multiple, serious, or major neurologic deficits, extensive systemic disease with few treatment options, bulky central nervous system (CNS) disease, or encephalopathy. Some exceptions to these symptoms, where a patient may still be considered for a more aggressive treatment course, include patients with exceptionally chemosensitive tumors (e.g., small cell lung cancer, lymphoma).

Traditionally, radiotherapy has been recommended for patients with symptomatic disease or bulky metastatic disease, regardless of clinical symptoms (4, 13, 15, 89–91). To date, there is no level I evidence available on the use of RT for LMD, as the only randomized controlled trial included in this systematic review sought to evaluate the use of ITC in patients with LMD from breast cancer (39). However, a large majority (69%) of the observational studies found an improvement or likely improvement in OS with the addition of RT to the treatment regimen of LMD of those studies that commented on the influence of a single or combined treatment regimen on survival (*n* = 18/26 studies).

To the best of our knowledge, this is the first systemic review that has attempted to assimilate the reported data for the use of radiotherapy in the treatment of LMD across a range histologies. In our own clinical experience, radiotherapy for the treatment of LMD is consistently considered and discussed in the multidisciplinary setting. The treatment of RT is often recommended for patients with bulky disease seen on imaging or causing symptoms to prevent further neurologic compromise, or for asymptomatic patients with LMD seen on MRI who have well-controlled extracranial disease.

The results of our review indicate that for the majority of studies, any treatment was felt to be superior to best supportive care in the management of LMD, but this may reflect a selection bias of patients with better prognosis and performance status to receive treatment. The use of RT monotherapy was found to be beneficial in patients with LMD from pediatric CNS gliomas, leukemia/lymphoma, gynecologic, esthesioneuroblastoma, and pediatric rhabdomyosarcoma histologies. For patients with LMD from breast cancer, melanoma and adult CNS cancer, multimodality therapy, including the use of radiotherapy, was found to be most beneficial. In terms of prognostic factors, MVA across the included series in this systematic review consistently revealed that a good performance status at the time of diagnosis of LMD and normal CSF flow predicted for improved survival. In patients with breast cancer, hormone receptor positive disease, limited prior systemic therapy exposure, and LMD as the first site of metastases additionally predicted for improved survival.

Due to the vast clinical and methodologic differences between the included studies, a meta-analysis could not be performed to identify the optimal treatments for patients with LMD. Most studies only reported whether RT was used or not, and what type of RT was used (WBRT, focal brain/spine RT, etc.) with a complete lack of details about RT dose, fields, or timing. Additionally, the role of targeted therapies and immunotherapy has greatly increased in the treatment of LMD in the past decade (91). For example, for NSCLC with LMD, current therapies target epidermal growth factor receptor (EGFR) mutations and anaplastic lymphoma kinase (ALK) rearrangement via tyrosine kinase inhibitors (TKIs); in breast cancer, HER2 positive disease may be targeted via an intrathecal injection; and finally, melanoma with BRAF V600E mutations may also be treated with targeted therapies. Given that the included studies only included patients treated through 2015, the role and impact of targeted therapies is not reflected in this systematic review. It will be imperative to re-evaluate the use of RT, paying special attention to the dose of RT, RT fields, and timing of RT, in conjunction with these targeted and immunotherapies, in future studies.

Additionally, new classifications of LMD are being reported that may influence survival outcomes and help aid in the most optimal therapy selection. A recent report by Prabhu et al. categorized the LMD pattern of their postoperative stereotactic radiosurgery cohort as nodular or the more classical “sugarcoating” pattern. (92) The authors found that nodular LMD was less likely to be symptomatic and had better OS outcomes. Furthermore, when the patients with nodular LMD were treated with focal RT, as opposed to WBRT, they had a higher risk of second LMD recurrence, but no detriment in OS, providing a reasonable, less toxic, treatment strategy for these patients. Unfortunately, LMD was not subclassified in the articles included in this review. Future studies should consider further subclassification of this heterogeneous disease.

### Limitations

In conducting this systematic review, it became very apparent that there is a paucity of prospective, interventional studies in the treatment of LMD, particularly for the use of radiotherapy for the treatment of LMD. The single RCT that met inclusion criteria was a randomized trial evaluating intraventricular chemotherapy for LMD that also reported that the use of RT with systemic chemotherapy was feasible in this population (39).

## Conclusions

In conclusion, LMD is a devastating complication of cancer with reported survivals from the time of diagnosis ranging from 2 to 4 months in prior literature (16–19), and 1–32 months in our review, depending on the primary histology. Based on this systematic review, the recommendation for the treatment of LMD is for multimodality discussion of cases and treatment, including the use of radiotherapy, for LMD. However, with continued advances in systemic therapy including targeted therapy and immunotherapy as well as imaging advances, the landscape of metastatic disease and LMD is evolving rapidly. Within this changing environment, the role of RT will likely also continue to evolve and advance. As this systematic review highlights, there is limited high-quality evidence to guide the optimal use of radiotherapy for the treatment of LMD, and there is a great need for prospective, histology specific investigation of the role of radiotherapy for LMD in the era of modern systemic therapies.

## Author Contributions

SB and CC reviewed the articles included in this systematic review. CC developed the concept of the review and reviewed the manuscript. SB wrote the manuscript.

### Conflict of Interest

The authors declare that the research was conducted in the absence of any commercial or financial relationships that could be construed as a potential conflict of interest.
