# Supplementary material for: Radiotherapy in Leptomeningeal Disease: A Systematic Review of Randomized and Non-randomized Trials
Source: Front Oncol. 2019 Nov 15;9:1224. doi: 10.3389/fonc.2019.01224 (PMC6872542; doi:10.3389/fonc.2019.01224)
Supplement: Supplementary file 1 [file Data_Sheet_1.pdf]

Appendix 1: Search Strategy: MEDLINE (Ovid)

1 MENINGEAL CARCINOMATOSIS/
2 MENINGEAL NEOPLASMS/sc
3 (leptomeningeal adj3 disease).ti,kf.*
4 (leptomenin adj3 (carcinoma* or metastas*)).ti,kf.*
5 (neoplastic adj3 meningiti).ti,kf.*
6 (meningeal adj3 (metastas* or carcinoma*)).ti,kf.*
7 (leptomening adj3 disseminat*).ti,kf.*
8 ((leptomeningeal adj3 disease) or (leptomenin* adj3 (carcinoma* or metastas*)) or (neoplastic adj3 meningiti*) or (leptomening* adj3 disseminat*) or (meningeal* adj3 (metastas* or carcinoma*))).ab.*
9 or/1-8 [LMD terms]
10 MENINGEAL CARCINOMATOSIS/rt
11 MENINGEAL NEOPLASMS/rt
12 exp RADIOTHERAPY/
13 RADIATION/
14 (radiotherap or radio-therap*).ti,kf.*
15 (radiat or irradiat* or reirradiat* or re-irradiat*).ti,kf.*
16 (radiosurg or radio-surg*).ti,kf.*
17 (chemoradiotherap or chemo-radiotherap* or radioimmunotherap* or radio-immunotherap*).ti,kf.*
18 WBRT.ti,kf.
19 (((leptomeningeal adj3 disease) or LMD or (leptomenin* adj3 (carcinoma* or metastas*)) or (neoplastic adj3 meningiti*) or (leptomening* adj3 disseminat*) or (meningeal* adj3 (metastas* or carcinoma*))) and (radiotherap* or radio-therap* or radiat* or irradiat* or reirradiat* or re-irradiat* or radiosurg* or radio-surg* or chemoradiotherap* or chemo-radiotherap* or radioimmunotherap* or radio-immunotherap*)).ab. [terms in abstract]*
20 or/10-19
21 9 and 20
22 exp TREATMENT OUTCOME/
23 SURVIVAL/
24 exp SURVIVAL ANALYSIS/
25 SURVIVAL RATE/
26 Kaplan-Meier.ab.
27 (surviv or mortalit* or outcome* or prognos*).ti,kf.*

28 (((leptomeningeal adj3 disease) or (leptomenin* adj3 (carcinomatos* or metastas*)) or (neoplastic adj3 meningiti*) or (leptomening* adj3 disseminat*) or (meningeal* adj3 (metastas* or carcinoma*))) and (surviv* or mortalit* or outcome* or prognos*)).ab.*
29 or/22-28
30 21 and 29 [LMD + RT + survival/outcome]
31 (animals not (human and animals)).sh.
32 30 not 31
33 limit 32 to english language
34 case reports.pt. or "case report".ti.
35 review.pt. or review.ti.
36 34 not 35 [case reports not part of a review]
37 33 and 36 [case reports]
38 33 not 37 [publications other than case reports]
