# Supplementary material for: Radiotherapy in Leptomeningeal Disease: A Systematic Review of Randomized and Non-randomized Trials
Source: Front Oncol. 2019 Nov 15;9:1224. doi: 10.3389/fonc.2019.01224 (PMC6872542; doi:10.3389/fonc.2019.01224)
Supplement: Supplementary file 2 [file Data_Sheet_2.pdf]

Appendix 2: Search Strategy: EMBASE (Ovid)

1 CARCINOMATOUS MENINGITIS/
2 MENINGEAL METASTASIS/
3 (leptomeningeal adj3 disease).ti,kw.*
4 (leptomenin adj3 (carcinoma* or metastas*)).ti,kw.*
5 (neoplastic adj3 meningiti).ti,kw.*
6 (meningeal adj3 (metastas* or carcinoma*)).ti,kw.*
7 (leptomening adj3 disseminat*).ti,kw.*
8 ((leptomeningeal adj3 disease) or LMD or (leptomenin* adj3 (carcinoma* or metastas*)) or (neoplastic adj3 meningiti*) or (leptomening* adj3 disseminat*) or (meningeal* adj3 (metastas* or carcinoma*))).ab. /freq=2*
9 or/1-8 [LMD terms]
10 CARCINOMATOUS MENINGITIS/rt
11 MENINGEAL METASTASIS/rt
*12 exp *CANCER RADIOTHERAPY/*
13 BRAIN RADIATION/
14 WHOLE BRAIN RADIOTHERAPY/
15 (radiotherap or radio-therap*).ti,kw.*
16 (radiat or irradiat* or reirradiat* or re-irradiat*).ti,kw.*
17 (radiosurg or radio-surg*).ti,kw.*
18 (chemoradiotherap or chemo-radiotherap* or radioimmunotherap* or radio-immunotherap*).ti,kw.*
19 WBRT.ti,kw.
20 (((leptomeningeal adj3 disease) or LMD or (leptomenin* adj3 (carcinomatos* or metastas*)) or (neoplastic adj3 meningiti*) or (meningeal* adj3 (metastas* or carcinomatos*))) adj10 (radiotherap* or radio-therap* or radiat* or irradiat* or reirradiat* or re-irradiat* or radiosurg* or radio-surg* or chemoradiotherap* or chemo-radiotherap* or radioimmunotherap* or radio-immunotherap*)).ab. [same "sentence" in abstract]*
21 or/10-20
22 9 and 21
23 exp TREATMENT OUTCOME/
24 exp SURVIVAL/
25 SURVIVAL ANALYSIS/
26 exp MORTALITY RATE/
27 CANCER MORTALITY/
28 Kaplan-Meier.ab.
29 (surviv or mortalit* or outcome* or prognos*).ti,kw.*

30 (((leptomeningeal adj3 disease) or LMD or (leptomenin* adj3 (carcinomatos* or metastas*)) or (neoplastic adj3 meningiti*) or (leptomening* adj3 disseminat*) or (meningeal* adj3 (metastas* or carcinoma*))) and (surviv* or mortalit* or outcome* or prognos*)).ab.*
31 or/23-30
32 22 and 31 [LMD + RT + survival]
33 (NONHUMAN/ or ANIMAL/ or ANIMAL EXPERIMENT/) not HUMAN/
34 32 not 33 [minus animal only]
35 limit 34 to english language
36 CASE REPORT/ or case report.ti.*
37 review.pt,ti.
38 36 not 37 [case reports not reviews]
39 35 and 38 [case reports]
40 35 not 39 [publications other then case reports]
41 limit 40 to conference abstract
42 40 not 41 [minus conference abstracts]
