# Supplementary material for: Radiotherapy in Leptomeningeal Disease: A Systematic Review of Randomized and Non-randomized Trials
Source: Front Oncol. 2019 Nov 15;9:1224. doi: 10.3389/fonc.2019.01224 (PMC6872542; doi:10.3389/fonc.2019.01224)
Supplement: Supplementary file 3 [file Data_Sheet_3.pdf]

Appendix 3: Search Strategy: Cochrane (Wiley)

#1 MeSH descriptor: [Meningeal Carcinomatosis] explode all trees
#2 MeSH descriptor: [Meningeal Neoplasms] explode all trees and with qualifier(s): [Secondary - SC]
#3 (leptomeningeal near/3 disease):ti,kw,ab*
#4 (leptomenin near/3 (carcinomatos* or metastas*)):ti,kw,ab*
#5 (neoplastic near/3 meningiti) .ti,kw,ab*
#6 (meningeal near/3 (metastas* or carcinomatos*)):ti,kw,ab*
#7 (leptomening near/3 disseminat*):ti,kw,ab*
#8 #1 or #2 or #3 or #4 or #5 or #6 or #7
#9 MeSH descriptor: [Meningeal Carcinomatosis] explode all trees and with qualifier(s): [Radiotherapy - RT]
#10 MeSH descriptor: [Meningeal Neoplasms] explode all trees and with qualifier(s): [Radiotherapy - RT]
#11 MeSH descriptor: [Radiotherapy] explode all trees
#12 MeSH descriptor: [Radiation Dosage] explode all trees
#13 MeSH descriptor: [Radiation Effects] explode all trees
#14 MeSH descriptor: [Radiation Exposure] explode all trees
#15 (radiotherap or radio-therap*):ti,kw,ab*
#16 (radiat or irradiat* or reirradiat* or re-irradiat*):ti,kw,ab*
#17 (radiosurg or radio-surg*):ti,kw,ab*
#18 (chemoradiotherap or chemo-radiotherap* or radioimmunotherap* or radio-immunotherap*):ti,kw,ab*
#19 (WBRT):ti,kw,ab
#20 #9 or #10 or #11 or #12 or #13 or #14 or #15 or #16 or #17 or #18 or #19
#21 #8 and #20
